# Development of a Preoperative Difficulty Score for Retroperitoneal Laparoscopic Adrenalectomy: A Single-Center Retrospective Study

**DOI:** 10.3390/jcm15166204

**Published:** 2026-08-11

**Authors:** Jinhu Chen, Cheng Zhang, Ligang Zhang, Hexi Du, Changsheng Zhan

**Affiliations:** 1 Department of Urology, The First Affiliated Hospital of Anhui Medical University, Hefei 230001, China; urologistcjh@fy.ahmu.edu.cn (J.C.); zhangligang@fy.ahmu.edu.cn (L.Z.); yfy1102539@fy.ahmu.edu.cn (H.D.); 2 Institute of Urology, Anhui Medical University, Hefei 230001, China; 13886477629@163.com; 3 Anhui Province Key Laboratory of Genitourinary Diseases, Anhui Medical University, Hefei 230001, China; 4 Department of Urology, General Hospital of Southern Theater Command, Guangzhou 510030, China

**Keywords:** adrenal tumor, laparoscopic adrenalectomy, surgical difficulty, perioperative factors, regression model, score system

## Abstract

**Background:** Retroperitoneoscopic laparoscopic adrenalectomy (RLA) is widely adopted for benign adrenal lesions due to its direct access and minimal bowel interference. However, expanding indications to larger tumors and complex anatomies increase technical difficulty because of the confined retroperitoneal space and obscured hilar landmarks. Current predictive models mainly focus on transperitoneal approaches and lack specificity for RLA. This study aimed to develop and internally validate a preoperative nomogram for predicting RLA-specific surgical difficulty. **Methods:** All patients undergoing RLA from April 2024 to February 2025 at a single center were included. Operative time and postoperative hemoglobin (Hb) reduction were used as surrogate markers of difficulty. Patients were classified into high- and standard-risk groups. Univariate and multivariate analyses were performed to identify predictors of difficult RLA and develop a composite difficulty score. **Results:** Tumor position (between the upper renal pole and renal pedicle: OR 8.819, 95% CI 3.981–21.462, *p* < 0.001) and pheochromocytoma (PHEO) pathology (OR 34.881, 95% CI 3.841–4719.428, *p* < 0.001) were the strongest independent predictors of high surgical difficulty, whereas tumor size (≥40 mm: OR 3.926, *p* = 0.071) showed limited predictive value. The composite score demonstrated excellent discrimination, with an optimism-corrected area under the curve (AUC) of 0.828 (95% CI 0.796–0.835). The nomogram showed robust calibration, and a cutoff score ≥ 2 yielded a negative predictive value of 0.940. **Conclusions:** This study developed an RLA-specific nomogram incorporating tumor size, position, and pathology. The tumor–renal pedicle relationship was more predictive of surgical difficulty than tumor size alone, providing risk stratification to guide surgical planning, optimize patient selection, and enhance safety as RLA indications expand. External validation is required to confirm its generalizability.

## 1. Introduction

Laparoscopic adrenalectomy has evolved into the gold standard for the management of small adrenal tumors, primarily owing to its favorable perioperative outcomes and minimally invasive profile [1,2,3,4]. While the transperitoneal approach offers a broader working space, RLA provides direct access to the adrenal gland without violating the peritoneal cavity, thereby minimizing bowel manipulation and facilitating a quicker return to daily activities [5,6].

Historically, the indications for RLA were restricted to small (<6 cm), benign lesions confined to the retroperitoneum [7]. However, with accumulating surgical expertise, contemporary indications have expanded to encompass larger tumors (up to 9 cm) and even select cases of suspected malignancy, provided there is no major vascular involvement or extensive local infiltration [7,8]. Despite the expanding adoption of RLA, the anatomical constraints of the retroperitoneal space impose distinct technical challenges. The restricted working envelope and scarcity of readily identifiable anatomical landmarks underlie its well-documented steeper learning curve and greater technical demands relative to the transperitoneal approach.

Consequently, to mitigate iatrogenic injury, shorten operative time, and reduce postoperative morbidity, precise preoperative risk stratification is essential. Several investigators have previously attempted to identify factors influencing the difficulty of adrenal surgery. Alberici et al. developed a preoperative “difficulty score” specifically for transabdominal adrenalectomy, incorporating variables such as tumor size and surgeon experience [9]. Similarly, Natkaniec et al. demonstrated that tumor size and malignant pathology were correlated with prolonged operative duration and excessive blood loss [10]. However, a dedicated scoring system tailored to the unique anatomical constraints of RLA remains lacking.

In this study, we retrospectively analyzed a cohort of patients who underwent RLA at our institution. We aimed to identify independent predictors of surgical difficulty and subsequently develop a robust preoperative nomogram to quantitatively predict the complexity of RLA.

## 2. Patients and Methods

### 2.1. Patient Population

**Figure 1:**
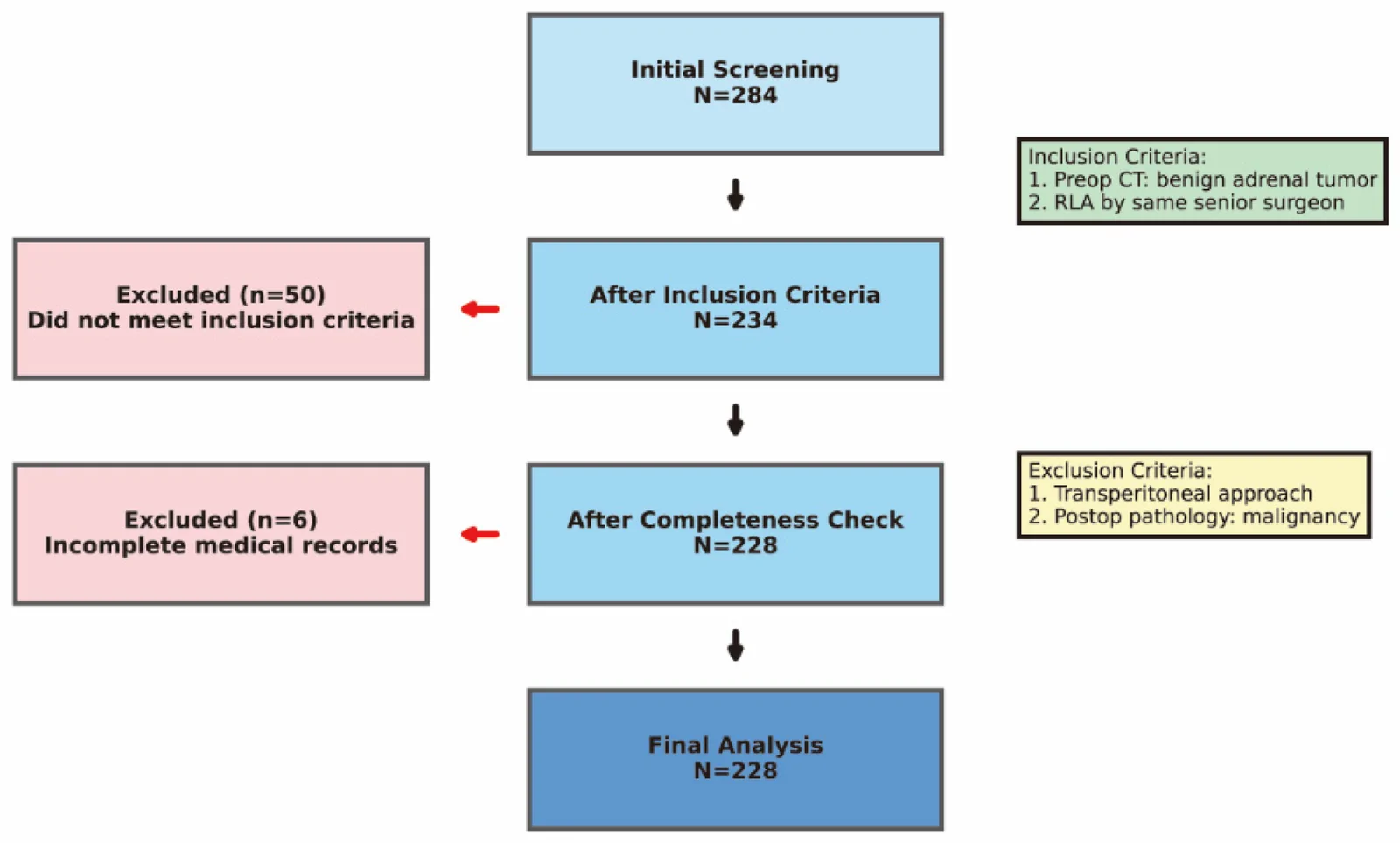
Patient flow diagram. A total of 284 patients who underwent RLA were initially screened, with 50 patients excluded after applying the eligibility criteria, and 6 were excluded due to incomplete medical records, leaving 228 patients included in the final analysis. Black arrows denote the progression of the study inclusion and exclusion process, whereas red arrows specifically indicate the removal of patient samples based on the predefined exclusion criteria.

A series of 284 patients who underwent RLA at the Department of Urology, The First Affiliated Hospital of Anhui Medical University, between April 2024 and February 2025 were initially screened, with 50 patients excluded for failing to meet the inclusion criteria, and 6 were excluded due to incomplete medical records, leaving 228 patients for final analysis (Figure 1).

Inclusion criteria: (1) Preoperative CT suggested a benign adrenal tumor. (2) Patients who underwent RLA performed by the same senior surgeon.

Exclusion criteria: (1) Patients who underwent transperitoneal laparoscopic adrenalectomy. (2) Patients with malignant tumors confirmed by postoperative pathology.

All procedures were performed by a specialized urological team. Preoperative assessments included contrast-enhanced CT and comprehensive biochemical evaluations to assess hormonal activity. This retrospective study was approved by the Institutional Ethics Review Board of Anhui Medical University, with written informed consent obtained from all participants prior to surgery.

### 2.2. Perioperative Measures and Imaging Analysis

Surgical difficulty was objectively quantified using two surrogate measures: operative time and postoperative Hb reduction. The 60 min threshold for classifying high surgical difficulty was chosen based on the cohort’s operative time distribution (median 70 min, IQR 55–91 min), corresponding to approximately the 40th percentile, balancing clinical relevance with model development needs. We systematically collected data on demographics (age, sex), anthropometrics (body mass index [BMI]), tumor characteristics (side, size, pathology), and critical anatomical distances measured on preoperative axial CT images: DAK represents the distance from the lower pole of the adrenal tumor to the upper renal pole. DARP represents the distance from the adrenal tumor to the renal pedicle. Tumor position was classified based on DAK and DARP values into three categories: above the renal upper pole, between the upper pole and renal pedicle, and below the renal pedicle. Inter-rater reliability for these measurements was assessed in 30 stratified cases by a second blinded urologist.

### 2.3. Surgical Procedure

**Figure 2:**
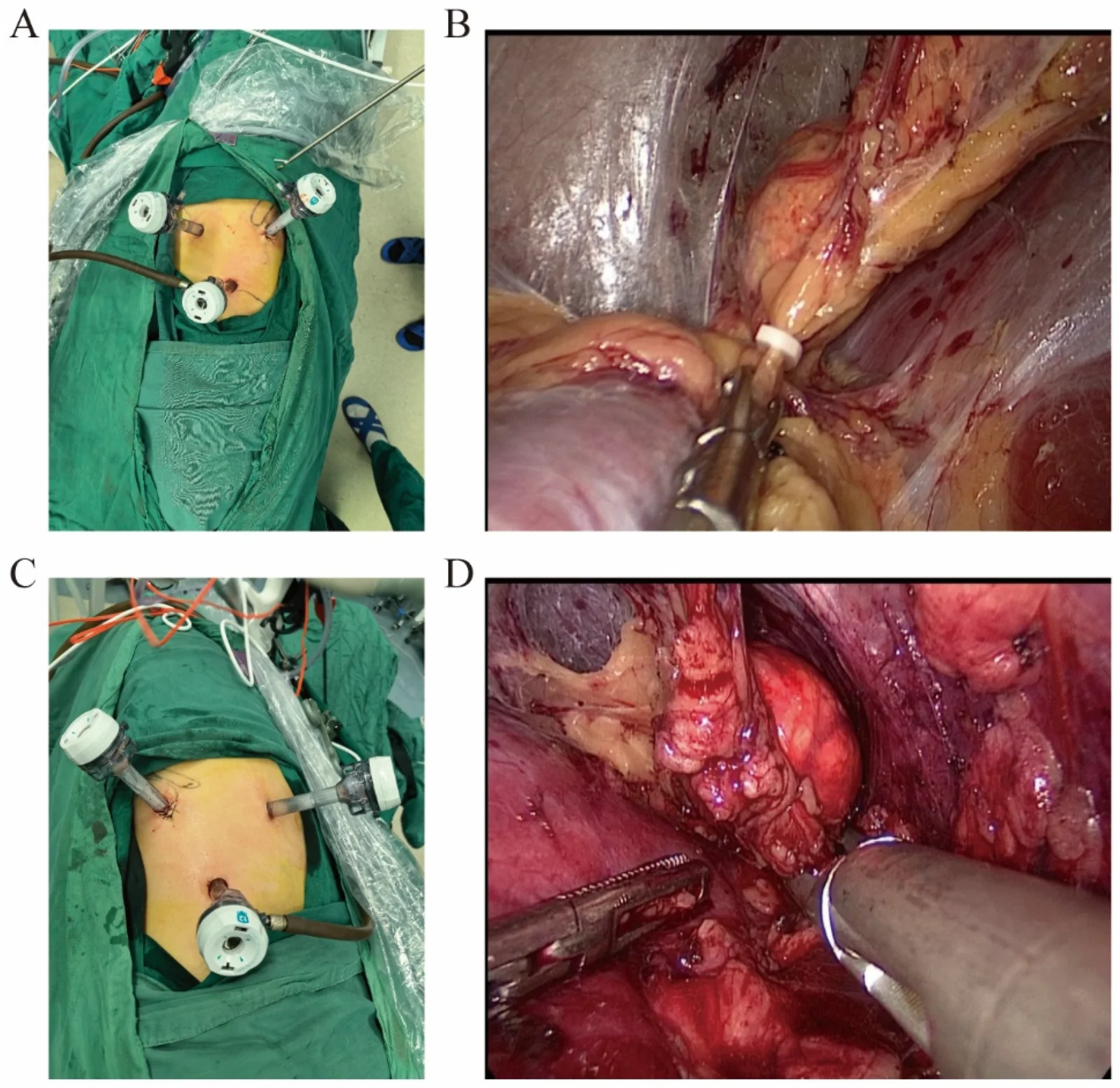
Trocar placement and intraoperative views during RLA. (**A**) Trocar placement for left-sided RLA. (**B**) Intraoperative view during left-sided RLA. (**C**) Trocar placement for right-sided RLA. (**D**) Intraoperative view during right-sided RLA.

Under general endotracheal anesthesia, patients were positioned laterally (affected side up, flexed). A 10 mm incision 2 cm below the costal margin (midaxillary line) was used to access the retroperitoneal space via balloon dilation (15 mmHg) and CO_2_ insufflation (12–15 mmHg) (Figure 2). Additional 5/10 mm trocars were placed laparoscopically. Retroperitoneal fat was dissected with an ultrasonic scalpel to expose Gerota’s fascia, which was incised for perirenal access. The adrenal gland (medial to the kidney and superior to the renal vein) was identified; adjacent structures were dissected. Vascular control included ligation of the central vein (3-0 polypropylene/hem-o-lok) and bipolar coagulation of arterial branches. The gland was circumferentially dissected with a 2–3 mm fat margin and removed via an endoscopic bag through the 10 mm port. Hemostasis was confirmed by irrigation; a 16-Fr Blake drain was placed. Operative time and postoperative Hb reduction were recorded; postoperative analgesia was administered as needed.

### 2.4. Statistical Analysis

Statistical analyses were executed using R (version 4.5.1). Continuous variables are presented as mean ± standard deviation (SD), while categorical variables are expressed as frequencies (percentages). Given that the continuous variables did not conform to a normal distribution, group comparisons were conducted using the Wilcoxon rank-sum test. Categorical variables were compared using the chi-square test. Linear regression was performed to explore associations between preoperative clinicopathological factors and surgical difficulty surrogates (operative time and postoperative Hb reduction). A composite difficulty score (range: 0–4) was subsequently constructed based on the 25th, 50th, 75th, and 90th percentiles of these surrogates, with a score ≥ 2 defined as the threshold for high surgical difficulty.

To identify independent predictors while mitigating bias from quasi-complete separation, Firth’s penalized likelihood logistic regression was implemented using the “logistf” package (version 1.26.1). Because “logistf” does not support the “reportROC” function for calculating sensitivity, specificity, PPV, NPV or the “validate” function, these diagnostic metrics were obtained from a standard “glm” sensitivity analysis without Firth correction, and bootstrap calibration was performed using a custom “rms”-based code. The regression coefficients derived from this model were used to construct a nomogram for individualized risk prediction using the “rms” package (version 8.0-0). Internal validation was conducted using 1000 bootstrap resamples to estimate the optimism-corrected AUC with a 95% confidence interval (CI). Calibration was assessed using calibration plots. A two-tailed *p* value < 0.05 was considered statistically significant. All visualizations were generated using the “ggplot2” package (version 3.5.2).

## 3. Results

### 3.1. General Characteristics of Study Cohort

The final analysis included 228 patients undergoing RLA. The baseline demographic, anthropometric, and radiological characteristics of the total cohort are summarized in Table 1. Briefly, the mean age was 50.88 years, and the majority of patients were female (57.5%). Pathologically, non-functional adrenal tumors (NFAT) were the most prevalent (42.1%), followed by primary aldosteronism (PA, 25.0%).

When stratified by operative time, no statistically significant differences were observed between the ≤60 min and >60 min groups regarding age, gender, BMI, tumor side, position, pathology, tumor size, or DARP (*p* > 0.05). Notably, DAK was significantly shorter in the prolonged operative time group (1.39 ± 9.55 mm vs. −1.69 ± 12.94 mm, *p* = 0.037), suggesting a potential association with surgical complexity. Inter-rater reliability analysis showed that the intraclass correlation coefficient (ICC) for DAK was 0.989 (95% CI: 0.977–0.995), the ICC for DARP was 0.990 (95% CI: 0.981–0.996), and Cohen’s κ value for position was 1.0 (*p* < 0.001).

### 3.2. Associations of Clinical Characteristics with Surgical Difficulty

To identify predictors of surgical difficulty, linear regression analyses were carried out using operative time and postoperative Hb reduction as the prespecified surrogate endpoints (Table 2 and Table 3).

Univariate linear regression identified tumor size as a positive correlate of operative time, whereas DAK and DARP exhibited significant inverse associations with surgical duration (*p* < 0.05). However, upon multivariable linear regression, only tumor size (β = 0.365, 95% CI: 0.033–0.697, *p* = 0.032) and DAK (β = −0.799, 95% CI: −1.152–(−0.446), *p* < 0.001) persisted as independently associated with prolonged surgery. Notably, DARP failed to retain significance in the multivariable analysis, suggesting that its apparent association with operative time in the univariate screen may be confounded by DAK. Other preoperative variables, including age, gender, BMI, tumor laterality, and pathological subtype, did not demonstrate independent predictive values for surgical duration (Table 2).

Parallel analyses were conducted for postoperative Hb reduction. Univariate screening identified associations between several factors and postoperative Hb reduction, including male gender, right-sided tumors, PHEO, “other” pathological types, tumor size, and DAK/DARP. Multivariable regression further clarified these relationships. Independent risk factors associated with greater postoperative Hb reduction included male sex (β = −1.912, 95% CI: −3.080–(−0.744), *p* = 0.002), right-sided laterality (β = −1.239, 95% CI: −2.433–(−0.044), *p* = 0.043), PHEO (β = 8.647, 95% CI: 5.660–11.635, *p* < 0.001), “other” pathological types (β = 1.929, 95% CI: 0.103–3.755, *p* = 0.040), and DARP (β = −0.176, 95% CI: −0.246–(−0.106), *p* < 0.001). In contrast to its role in predicting operative time, DAK was not an independent predictor of Hb loss in the adjusted model, suggesting that DARP may represent a more relevant anatomical factor associated with intraoperative blood loss (Table 3).

### 3.3. Development and Validation of the Difficulty Score

To operationalize surgical difficulty, a composite scoring system was first established using the percentile distributions of two objective procedural surrogates: operative time and postoperative Hb reduction (Table 4). Weighted integer scores (0–2) were assigned to each parameter based on the 25th, 50th, 75th, and 90th percentiles, generating a cumulative difficulty score ranging from 0 to 4 per patient. A cut-off value of ≥2 was selected to dichotomize surgical difficulty, as this threshold balanced a high sensitivity (0.837, 95% CI: 0.733–0.940) with an excellent negative predictive value (0.940, 95% CI: 0.900–0.980), thereby reducing the misclassification of high-difficulty procedures as standard-risk cases (Table 5).

**Figure 3:**
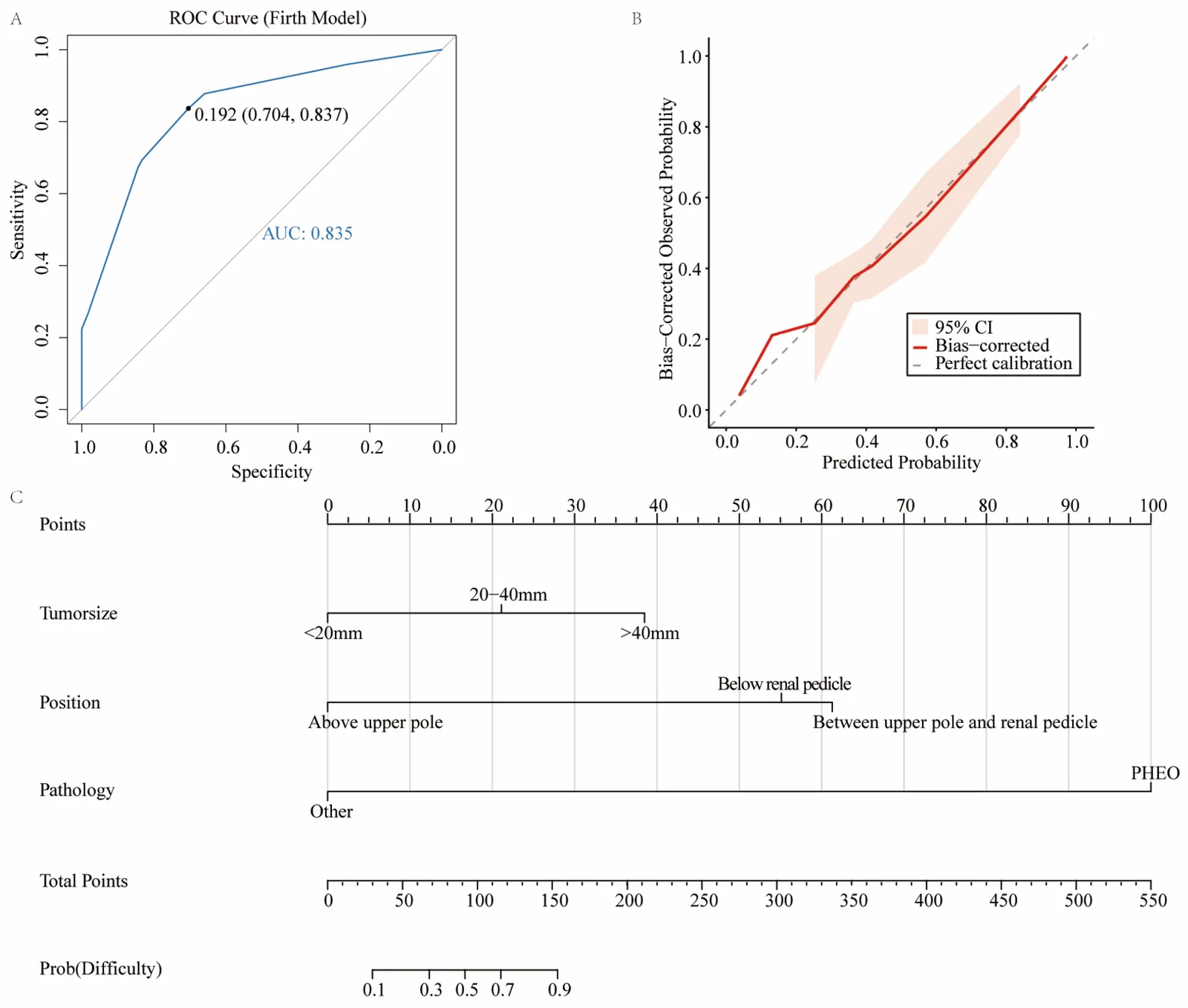
Development and internal validation of the surgical difficulty prediction model. (**A**) Receiver operating characteristic (ROC) curve of the Firth penalized logistic regression model. The area under the curve (AUC) was 0.835, indicating excellent discrimination. (**B**) Calibration plot of the model based on 1000 bootstrap resamples, demonstrating good agreement between predicted and observed probabilities (Note: The model converged in 994 of 1000 bootstrap samples). (**C**) Clinical nomogram for estimating the individualized probability of high surgical difficulty. Total points are calculated by summing the scores corresponding to tumor size, tumor position, and pathology. The total points are projected vertically to the bottom scale to obtain the predicted probability of high surgical difficulty.

Subsequently, Firth penalized likelihood logistic regression was used to identify independent predictors of the dichotomized outcome (Table 6). This method corrected bias caused by quasi-complete separation, which may occur in cohorts with low event counts. Tumor position was a key anatomical predictor. Lesions located between the renal upper pole and renal pedicle had significantly higher odds of difficulty than those located above the upper pole (OR = 8.819, 95% CI: 3.981–21.462, *p* < 0.001). Pathology was the strongest predictor, with PHEO conferring substantially higher odds of difficulty relative to non-PHEO entities (OR = 34.881, 95% CI: 3.841–4719.428, *p* < 0.001). Larger tumor size also demonstrated a dose–response relationship and was retained in the model despite not reaching conventional statistical significance, owing to its established clinical relevance. Internal validation via 1000 bootstrap resamples confirmed the model’s stability (convergence rate: 994/1000). The Firth logistic regression model demonstrated excellent discrimination, with an apparent AUC of 0.835, which remained robust after bias correction (optimism-corrected AUC = 0.828, 95% CI: 0.796–0.835; Figure 3A). Furthermore, the bootstrap-calibrated curve revealed a close agreement between predicted probabilities and observed outcomes across the full risk spectrum, indicating robust model calibration (Figure 3B).

Finally, to translate these statistical findings into a clinically accessible instrument, a nomogram was constructed based on the regression coefficients derived from the Firth-adjusted model (Figure 3C). This tool allows for individualized estimation of individualized surgical difficulty risk by integrating tumor size, position, and pathology into a summed total points axis, which directly corresponds to a predicted probability of encountering high surgical difficulty.

## 4. Discussion

RLA has gained widespread acceptance for benign adrenal lesions owing to its direct access to the adrenal gland without traversing the peritoneal cavity. However, preoperative risk stratification for RLA remains less standardized compared with its transperitoneal counterpart. Several scoring systems have been proposed to predict adrenal surgical difficulty, which primarily incorporate tumor size, pathology and surgeon experience [9,10]. More recent modifications have added radiographic parameters such as “medialness” or distance from vessels [11]. In this study, a novel difficulty score specifically tailored for RLA was developed and internally validated. This system utilizes operative time and postoperative Hb reduction as dual surrogate measures, while incorporating tumor location relative to the kidney and renal pedicle, tumor size, and pathological type as primary predictors. The Firth penalized logistic regression model, selected to mitigate quasi-complete separation arising from limited group sizes, yielded an optimism-corrected AUC of 0.828 (95% CI: 0.796–0.835), supporting robust discriminative performance.

An important finding of this study is that the anatomical relationship between the adrenal tumor and the kidney, particularly the proximity to the renal pedicle, showed stronger predictive value than tumor size for surgical difficulty. While tumor size ≥ 40 mm showed a positive trend (OR 3.926, *p* = 0.071), lesions located between the upper renal pole and the renal pedicle were associated with an approximately nine-fold increase in the odds of difficulty (OR 8.819, *p* < 0.001). This observation is biologically and technically plausible. In RLA, the retroperitoneal working space is inherently narrow, and the surgeon’s view of the adrenal central vein and renal pedicle is often obstructed by the upper renal pole itself [12]. As the tumor descends toward the pedicle (“upper renal pole” and “Renal pedicle” categories defined in this classification), the dissection plane migrates from the relatively avascular pericapsular space into the hilar zone, where the inferior vena cava, renal vein, and early arterial branches demand meticulous control. Prior studies on transperitoneal laparoscopic adrenalectomy have similarly noted that tumors “below the upper pole” or with medial extension increase conversion rates [11], but these nuances have been under-explored in RLA-specific cohorts. This study suggests that for RLA, the critical consideration is not merely tumor size, but rather the extent of inferior tumor extension along the renal pedicle axis-a distinction that refines preoperative counseling and may guide the choice between retroperitoneal and transperitoneal approaches in borderline cases.

Consistent with prior reports [13,14], PHEO emerged as the strongest independent predictor (OR 34.881, *p* < 0.001). The hypervascularity of PHEO, coupled with the risk of catecholamine-induced hemodynamic instability during manipulation, may increase procedural complexity regardless of tumor dimensions, a finding that reinforces the inclusion of pathological factors, not merely imaging traits, in difficulty scoring systems. Methodologically, Firth’s penalized likelihood regression was adopted to address potential quasi-complete separation, representing a technique increasingly advocated for clinical modeling with sparse event data [15,16]. Coupled with 1000 bootstrap resamples for optimism correction, this approach may reduce the risk of overfitting—a common critique of prediction models derived from single-center retrospective data. The high negative predictive value (NPV 0.940) of ≥2 cut-off further implies that low-scoring patients may be considered at lower risk preoperatively with minimal risk of “hidden” difficulty.

The nomogram, which integrates tumor size, position, and pathology into an individualized risk probability, serves as a pragmatic clinical tool for surgeons. Crucially, tumor position can be readily determined through visual inspection of routine preoperative CT scans without necessitating complex geometric measurements or advanced post-processing. This simplicity enhances the model’s clinical applicability and facilitates rapid preoperative decision-making. For centers where RLA is the preferred approach for right-sided or smaller lesions, this score aids in identifying candidates at risk of conversion or excessive blood loss, thereby prompting earlier preparation. Furthermore, as the indications for RLA expand beyond small, peripherally located lesions to include larger tumors or those approaching the renal pedicle [7], the ability to preoperatively identify high-risk anatomy becomes crucial. Our nomogram provides a quantitative framework to support this expansion, supporting surgeons in selecting appropriate candidates for RLA while identifying those who may derive greater benefit from a transperitoneal approach or open conversion.

Limitations include the single-center, retrospective design and the fact that all procedures were performed by a single senior surgeon, which may limit generalizability to junior operators or other institutional workflows. External validation across multi-surgeon, multi-center cohorts is warranted, specifically encompassing domestic multi-surgeon trials, international Western-center collaboration, and enriched PHEO enrollment to narrow the wide confidence interval. Additionally, our composite endpoint includes operative time and postoperative Hb reduction to reflect technical and hemostatic difficulty. Postoperative complications are not directly captured by this composite measure. The Clavien–Dindo classification will be integrated into future analyses. It is also worth noting that while our model aids in patient selection, the expanding indications for RLA—such as tumors exceeding 6 cm—warrant cautious interpretation. Future external validation should specifically assess whether this scoring system retains its predictive accuracy in these more challenging subsets of patients, where the balance between oncological safety and surgical feasibility is more delicate.

In summary, we propose a preoperative difficulty score for RLA that prioritizes tumor position along the renal pedicle axis and PHEO pathology alongside tumor size. By anchoring risk stratification in retroperitoneal anatomy rather than size alone, this tool refines case selection and anticipatory planning for adrenal surgery. However, prospective external validation remains imperative to corroborate the model’s utility across diverse patient populations.

## Figures and Tables

**Table 1:** Characteristics of the study cohort and comparison between operative time groups.

Variable	Total Cohort	Operative Time		*p* Value
	(*n* = 228)	≤60 min (*n* = 80)	>60 min (*n* = 148)	
Age	50.88 ± 12.47	49.98 ± 12.88	51.36 ± 12.26	0.424
Gender				0.158
Female	131 (57.5)	51 (63.7)	80 (54.1)	
Male	97 (42.5)	29 (36.2)	68 (45.9)	
BMI (kg/m^2^)	24.46 ± 2.36	24.45 ± 2.29	24.47 ± 2.41	0.895
Side				0.063
Left	112 (49.1)	46 (57.5)	66 (44.6)	
Right	116 (50.9)	34 (42.5)	82 (55.4)	
Position				0.272
Above upper pole	135 (59.2)	53 (66.2)	82 (55.4)	
Between upper pole and renal pedicle	88 (38.6)	26 (32.5)	62 (41.9)	
Below renal pedicle	5 (2.2)	1 (1.2)	4 (2.7)	
Pathology				0.715
NFAT	96 (42.1)	37 (46.2)	59 (39.9)	
PA	57 (25.0)	20 (25.0)	37 (25.0)	
CS	26 (11.4)	10 (12.5)	16 (10.8)	
PHEO	11 (4.8)	3 (3.8)	8 (5.4)	
Other	38 (16.7)	10 (12.5)	28 (18.9)	
Tumor Size (mm)	24.72 ± 11.53	23.24 ± 8.52	25.51 ± 12.82	0.396
DAK (mm)	−0.61 ± 11.93	1.39 ± 9.55	−1.69 ± 12.94	0.037 *
DARP (mm)	29.11 ± 11.08	29.38 ± 10.68	28.97 ± 11.32	0.894

Data are presented as mean ± standard deviation (SD) for continuous variables and *n* (%) for categorical variables. Continuous variables were compared using the Wilcoxon rank-sum test, and categorical variables were analyzed using the χ^2^ test. Abbreviations: BMI, body mass index; NFAT, non-functional adrenal tumor; PA, primary aldosteronism; CS, Cushing syndrome; PHEO, pheochromocytoma; DAK, distance from the lower pole of the adrenal tumor to the upper renal pole; DARP, distance from the adrenal tumor to the renal pedicle. Other pathological types included myelolipoma, cyst, ganglioneuroma, lymphangioma, and teratoma. Statistical significance was defined as *p* < 0.05. Significance levels: * *p* < 0.05.

**Table 2:** Univariate and multivariable linear regression analyses of factors associated with operative time.

	Univariate			Multivariate		
	β	95% CI	*p* Value	β	95% CI	*p* Value
Age	0.074	−0.194–0.343	0.589	0.061	−0.200–0.322	0.649
Gender						
Female	Ref.	-	-	Ref.	-	-
Male	6.221	−0.503–12.945	0.071	5.315	−1.226–11.855	0.113
BMI (kg/m^2^)	−0.026	−1.446–1.392	0.971	−0.341	−1.687–1.005	0.620
Side						
Left	Ref.	-	-	Ref.	-	-
Right	0.894	−5.803–7.591	0.794	3.667	−3.022–10.356	0.284
Pathology						
NFAT	Ref.	-	-	Ref.	-	-
PA	−4.049	−12.485–4.387	0.348	−2.116	−10.349–6.117	0.615
CS	−6.185	−17.339–4.969	0.278	−6.308	−17.220–4.605	0.258
PHEO	9.969	−6.090–26.028	0.225	−1.838	−18.565–14.89	0.830
Other	−0.110	−9.779–9.559	0.982	−4.631	−14.855–5.593	0.376
Tumor Size (mm)	0.394	0.107–0.680	0.008 **	0.365	0.033–0.697	0.032 *
DAK (mm)	−0.725	−0.990–(−0.460)	<0.001 ***	−0.799	−1.152–(−0.446)	<0.001 ***
DARP (mm)	−0.351	−0.651–(−0.052)	0.022 *	0.179	−0.213–0.572	0.372

Data are presented as regression coefficients (β) with 95% confidence intervals (CIs). Ref., reference category. “Other” pathological types included myelolipoma, ganglioneuroma, lymphangioma, teratoma, and adrenal cysts. Abbreviations: BMI, body mass index; NFAT, non-functional adrenal tumor; PA, primary aldosteronism; CS, Cushing syndrome; PHEO, pheochromocytoma; DAK, distance from the lower pole of the adrenal tumor to the upper renal pole; DARP, distance from the adrenal tumor to the renal pedicle. Significance levels: * *p* < 0.05, ** *p* < 0.01, *** *p* < 0.001.

**Table 3:** Univariate and multivariable linear regression analyses of factors associated with postoperative Hb reduction.

	Univariate			Multivariate		
	β	95% CI	*p* Value	β	95% CI	*p* Value
Age	−0.026	−0.083–0.030	0.361	−0.011	−0.057–0.036	0.651
Gender						
Female	Ref.	-	-	Ref.	-	-
Male	−1.987	−3.383–(−0.590)	0.006 **	−1.912	−3.080–(−0.744)	0.002 **
BMI (kg/m^2^)	0.053	−0.245–0.350	0.729	0.035	−0.205–0.276	0.773
Side						
Left	Ref.	-	-	Ref.	-	-
Right	−1.420	−2.813–(−0.027)	0.047 *	−1.239	−2.433–(−0.044)	0.043 *
Pathology						
NFAT	Ref.	-	-	Ref.	-	-
PA	0.065	−1.505–1.636	0.935	0.480	−0.990–1.950	0.523
CS	−0.198	−2.275–1.879	0.852	−1.026	−2.974–0.923	0.304
PHEO	11.777	8.787–14.767	<0.001 ***	8.647	5.660–11.635	<0.001 ***
Other	1.979	0.178–3.779	0.032 *	1.929	0.103–3.755	0.040 *
Tumor Size (mm)	0.110	0.051–0.169	<0.001 ***	0.050	−0.009–0.109	0.099
DAK (mm)	−0.121	−0.178–(−0.065)	<0.001 ***	0.026	−0.037–0.089	0.411
DARP (mm)	−0.221	−0.278–(−0.165)	<0.001 ***	−0.176	−0.246–(−0.106)	<0.001 ***

Data are presented as regression coefficients (β) with 95% confidence intervals (CIs). Ref., reference category. “Other” pathological types included myelolipoma, ganglioneuroma, lymphangioma, teratoma, and adrenal cysts. Abbreviations: BMI, body mass index; NFAT, non-functional adrenal tumor; PA, primary aldosteronism; CS, Cushing syndrome; PHEO, pheochromocytoma; DAK, distance from the lower pole of the adrenal tumor to the upper renal pole; DARP, distance from the adrenal tumor to the renal pedicle. Significance levels: * *p* < 0.05, ** *p* < 0.01, *** *p* < 0.001.

**Table 4:** Assignment of the composite difficulty score based on percentile thresholds of operative time and postoperative Hb reduction.

	P25	P50	P75	P90
Operative time (min)	55	70	91	115
postoperative Hb reduction (g/L)	5.68	10.25	12.50	16.20
Points	0		1	2

Scores range from 0 to 2 for each variable, yielding a total composite difficulty score ranging from 0 to 4. Abbreviation: Hb, hemoglobin.

**Table 5:** Diagnostic performance of the composite difficulty score for identifying high surgical difficulty.

Score	Sensitivity	Specificity	PPV	NPV	AUC
≥1	0.657 (0.565–0.749)	0.786 (0.714–0.857)	0.713 (0.621–0.804)	0.739 (0.664–0.813)	0.757 (0.695–0.819)
≥2	0.837 (0.733–0.940)	0.704 (0.637–0.771)	0.436 (0.336–0.536)	0.940 (0.900–0.980)	0.835 (0.771–0.900)
≥3	0.818 (0.590–1.000)	0.747 (0.689–0.804)	0.141 (0.055–0.226)	0.988 (0.971–1.005)	0.832 (0.723–0.942)
≥4	0.667 (0.289–1.000)	0.968 (0.945–0.991)	0.364 (0.079–0.648)	0.991 (0.978–1.003)	0.863 (0.683–1.000)

Values are presented for various cutoff points defining high surgical difficulty. Diagnostic indices were obtained from a sensitivity analysis using standard logistic regression without Firth correction. Data are presented as model estimates with 95% confidence intervals (CIs); no optimism correction was applied. Abbreviations: AUC, area under the receiver operating characteristic curve; PPV, positive predictive value; NPV, negative predictive value.

**Table 6:** Firth penalized likelihood logistic regression analysis of independent predictors associated with high surgical difficulty.

	β	OR	95% CI	*p* Value
Tumor Size				
<20 mm	Ref.	-	-	-
20–40 mm	0.750	2.116	0.911–5.254	0.082
≥40 mm	1.368	3.926	0.885–16.675	0.071
Position				
Above upper pole	Ref.	-	-	-
Between upper pole and renal pedicle	2.177	8.819	3.981–21.462	<0.001 ***
Below renal pedicle	1.957	7.080	0.510–62.452	0.125
Pathology				
None-PHEO	Ref.	-	-	-
PHEO	3.552	34.881	3.841–4719.428	<0.001 ***

Firth’s penalized likelihood method was applied to reduce bias caused by quasi-complete separation. Abbreviations: OR, odds ratio; CI, confidence interval; PHEO, pheochromocytoma. Significance levels: *** *p* < 0.001.

## Data Availability

Due to the sensitive nature of the data and confidentiality commitments to participants, the dataset is not publicly available but can be requested from the corresponding author.
